# Nuclear Multidrug Resistance-Related Protein 1 Is Highly Associated with Better Prognosis of Human Mucoepidermoid Carcinoma through the Suppression of Cell Proliferation, Migration and Invasion

**DOI:** 10.1371/journal.pone.0148223

**Published:** 2016-02-01

**Authors:** Bo-Lei Cai, Yan Li, Liang-Liang Shen, Jin-Long Zhao, Yuan Liu, Jun-Zheng Wu, Yan-Pu Liu, Bo Yu

**Affiliations:** 1 State Key Laboratory of Military Stomatology, Department of Oral and Maxillofacial Surgery, School of Stomatology, the Fourth Military Medical University, Xi’an 710032, People's Republic of China; 2 State Key Laboratory of Military Stomatology, Department of Prosthodontics, School of Stomatology, the Fourth Military Medical University, Xi'an 710032, People's Republic of China; 3 The State Laboratory of Cancer Biology, Department of Biochemistry and Molecular Biology, the Fourth Military Medical University, Xi’an 710032, People's Republic of China; 4 State Key Laboratory of Military Stomatology, Department of Oral Histology and Pathology, School of Stomatology, the Fourth Military Medical University, Xi’an 710032, People's Republic of China; 5 State Key Laboratory of Military Stomatology, Department of Oral Biology, School of Stomatology, The Fourth Military Medical University, 145 Chang Le Xi Road, Xi’an 710032, People's Republic of China; Georgetown University, UNITED STATES

## Abstract

**Objectives:**

Multidrug resistance-related protein 1 (MRP1) overexpression is a well acknowledged predictor of poor response to chemotherapy, but MRP1 also correlated to better prognosis in some reports, especially for patients not pretreated with chemotherapy. In our previous study, we found nuclear translocation of MRP1 in mucoepidermoid carcinoma (MEC) for the first time. The purpose of this study was to further investigate the function of nuclear MRP1 in MEC.

**Materials and Methods:**

Human MEC tissue samples of 125 patients were selected and stained using immunohistochemistry. The expression level of total MRP1/nuclear MRP1 of each sample was evaluated by expression index (EI) which was scored using both qualitative and quantitative analysis. The correlations between the clinicopathologic parameters and the EI of nuclear MRP1 were analyzed using Spearman’s rank correlation analysis, respectively. The effects of RNAi-mediated downregulation of nuclear MRP1 on MEC cells were assessed using flow cytometric analysis, MTT assay, plate colony formation assay, transwell invasion assay and monolayer wound healing assay.

**Results:**

In this study, we found the EI of nuclear MRP1 was negatively correlated to the pathologic grading (r = -0.498, P<0.01) / clinical staging (r = -0.41, P<0.01) / tumor stage (r = -0.28, P = 0.02) / nodal stage (r = -0.29, P<0.01) of MEC patients. The RNAi-mediated downregulation of nuclear MRP1 further proved that the downregulation of nuclear MRP1 could increase the cell replication, growth speed, colony formation efficiency, migration and invasion ability of MEC cells.

**Conclusion:**

Our results suggested that nuclear MRP1 is highly associated with better prognosis of human mucoepidermoid carcinoma and further study of its function mechanism would provide clues in developing new treatment modalities of MEC.

## Introduction

Multidrug resistance-related protein 1 (MRP1 or ABCC1), which is an energy-dependent transporter, was first discovered in a multidrug-resistant small-cell lung cancer cell line [1]. It has been generally accepted that overexpression of MRP1 is a predictor of poor response to chemotherapy in a variety of hematological and solid tumors [2]. Nevertheless, for patients not pretreated with chemotherapy, MRP1 overexpression means better prognosis [3,4]. Despite the comprehensive knowledge of MRP1, the crystal structure and the transport mechanism of MRP1/ABCC1 remains elusive [5], especially when polymorphisms and mutations of MRP1 broadly existed in tumors [6,7].

Mucoepidermoid carcinoma (MEC) is the most common primary oral and maxillofacial malignant salivary gland tumor in the Chinese population [8,9]. It comprises about 35% of malignant salivary gland lesions. At present, the treatment plan of MEC is mainly based on pathologic grading. According to the morphological and cytological features, the pathologic grade of MEC is divided into low, intermediate and high grade MEC. The high-grade MEC patients have a very poor prognosis with a 5-year survival rate of only about 30%, yet the prognosis of low/intermediate-grade MEC patients is comforting [10,11]. Nonetheless, in some low/intermediate-grade MEC cases, destruction of adjacent bones, multiple local recurrences, regional lymph nodes metastases and distant metastases were found. Complete resection of primary lesion is the standard treatment of MEC [12]. However, Leverstein et.al pointed out that patients with MEC generally carried a high risk of lymph nodes metastasis [13] and the presence of lymph nodes metastases was the strongest prognostic factor of treatment failure [13,14]. We can treat high grade MEC aggressively because of its high incidence of lymph nodes metastasis. However, the decision to whether to perform neck dissection aggressively on low and intermediate grade MEC patients has persisted in perplexing the doctors.

In our previous study, we proved that nuclear translocation of MRP1 contributed to multidrug-resistance of mucoepidermoid carcinoma (MEC) via modulating the activity of multidrug resistance protein 1 (MDR1) promoter [15,16]. In this study, we found the nuclear MRP1 was highly related to the pathologic grading and the clinical staging of MEC. The downregulation of nuclear MRP1 enhanced the proliferation, migration and invasion of MEC cells in vitro. All our results suggested that the nuclear MRP1 might be a promising biomarker in predicting prognosis of MEC patients.

## Materials and Methods

### 2.1 Patient materials

Four normal salivary gland tissues were obtained from the Department of Oral and Maxillofacial Surgery, School of Stomatology, the Fourth Military Medical University; Human MEC tissues were obtained from 125 patients who had received no pretreatment before surgeries at the Department of Oral and Maxillofacial Surgery, School of Stomatology, the Fourth Military Medical University, from July 2006 to July 2011. The study group of the MEC patients included 54 males (43.2%) and 71 females (56.8%), with a mean age of 39 years (ranging from 1 year to 78 years). 64 patients (51.2%) had the primary tumors on major glands (parotid gland, submandibular gland and sublingual gland), and 61 patients (48.8%) on minor glands. After being formalin-fixed, paraffin-embedded, the specimens were diagnosed and identified by the Department of Oral Histology and Pathology, School of Stomatology, Fourth Military Medical University. Each tumor was pathologically graded according to a well accepted system which uses five pathologic features as the criteria [11,17]. According to the pathologic and clinical reports, the clinical stages (TNM system) were classified according to the 7th edition of the AJCC cancer staging manual [18]. The protocol and performance of our research was approved and monitored by the Ethics Committee of the Fourth Military Medical University (approval number: PLA, JRB-REV-201217). All patients involved have been informed of the research policies and signed the consent for their participation in this research. For the minors/children patients, written informed consent was signed by kin or guardians on their behalf.

### 2.2 Immunohistochemistry and the assessment of immunostaining

The immunohistochemistry staining was performed as previously described [15]. To assess the immunostaining and perform statistical analysis, the expression index (EI) of total MRP1/nuclear MRP1 was scored from 0 to 9 as previously described [15].

### 2.3 Cell culture

The expression of MRP1 is down-regulated by short-hairpin RNA (shRNA). The multidrug-resistant MC3/5FU cells were transfected with plasmids containing an MRP1 specific shRNA and a non-specific control shRNA, the resulting clones were MC3/5FU—S and MC3/5FU-NS. MC3/5FU and its transfected stable clones MC3/5FU—S cells and MC3/5FU-NS cells were verified and cultured as previously described (23).

### 2.4 Measurement of cell growth by methyl thiazolyl tetrazolium assay (MTT assay)

Cells were inoculated into 96-well plates at a density of 1×10^3^ cells/well and incubated for eight days. Six wells from each group were randomly selected and measured using MTT assay as previously described [16].

### 2.5 Flow cytometric analysis of the cell cycle

The cells were seeded in 25 ml flasks and incubated until they were 80–85% confluent. Then the cells were harvested, washed twice with ice-cold PBS, fixed with 70% ethanol overnight at 4°C, washed and resuspended in 100 μl of PBS containing a final concentration of 50 μg/ml RNase A for 30 minutes at room temperature. Finally, the cells were stained with 20 μg/ml PI in a final volume of 300μl for 20 minutes. DNA content and cell cycle were analyzed with a flow cytometer (BD-LSR) using CellQuest software. For each sample, a minimum of 10,000 cells were collected and counted.

### 2.6 Plate colony formation assay

For colony formation assays, 1x10^3^ cells were inoculated into 60mm dishes with 5ml RPMI1640 supplemented with 10% FBS. After 14 days, the resulting colonies were rinsed with PBS, and then fixed with 4% formaldehyde for 10 min, and stained with Giemsa (Sigma, USA) for 40 minutes, then rinsed with PBS again. Only the visible colonies (diameter>50μm) were counted.

### 2.7 Transwell invasion assay

The cells starved in serum-free RPMI1640 medium for 24 hours were prepared. Briefly, transwell inserts chambers (Becton Dickinson, Franklin Lakes, NJ) with 8 μm-pore filters were coated with Matrigel (Becton Dickinson, Bedford, MA, USA) of final concentration of 1 mg/ml of. Cells with a density of 1×10^4^ cells/ml were cultured on the upper chambers with 200 μl serum-free RPMI1640 medium and the lower wells were filled with 500μl RPMI1640 with 10% FBS as an inducer of cell migration. Cells continued migrating for 24 hrs. Cells on the filter were fixed with 4% formaldehyde and cells that remained on the upper surface of the filter would be removed using cotton swabs. After fixing for 15 minutes at room temperature, the chambers were rinsed in PBS and stained with Giemsa (Sigma, USA) for 5 minutes. The cells that migrated to the lower surface of the filter were examined by microscope after being mounted on a slide. A total of six random high-power microscopic fields (HPF) (100×) per filter were photographed. Then the numbers of cells were counted.

### 2.8 Monolayer wound healing assay

Cells were seeded into the 60mm cell culture plates until they were 90% confluent. Then the medium was replaced with FBS-free medium and incubated for 24 hours. A sterile 200μl pipette tip was used for creating a wound in the monolayer by scraping. The cells were washed with PBS and grown in FBS-free medium for a further 24 hours. The wounds were observed under a Leica DMI6000 B Fully Automated Inverted Research Microscope (Leica Microsystems, Germany). The width of the scratch was respectively measured at 0 and at 24 hour post-treatment. The migration distance in the wound was calculated by the formula below: cell free area at 0 hour—cell free area at 24 hour.

### 2.9 Statistical analysis

All data were expressed as means ± standard error (SEM). Student’s t-test and one-way ANOVA (LSD) were used for determining the significance of difference in comparisons. The relationship between the EI of total MRP1/ nuclear MRP1 and pathologic grade/clinical stage was analyzed with Spearman’s rank correlation analysis. The correlation coefficient (r) was calculated to measure the correlation degree. Calculations were carried out by software SPSS version 12.0, P<0.05 was considered statistically significant.

## Results

### 3.1 The expression of nuclear MRP1 is highly associated with MEC pathologic grading

**Fig 1:**
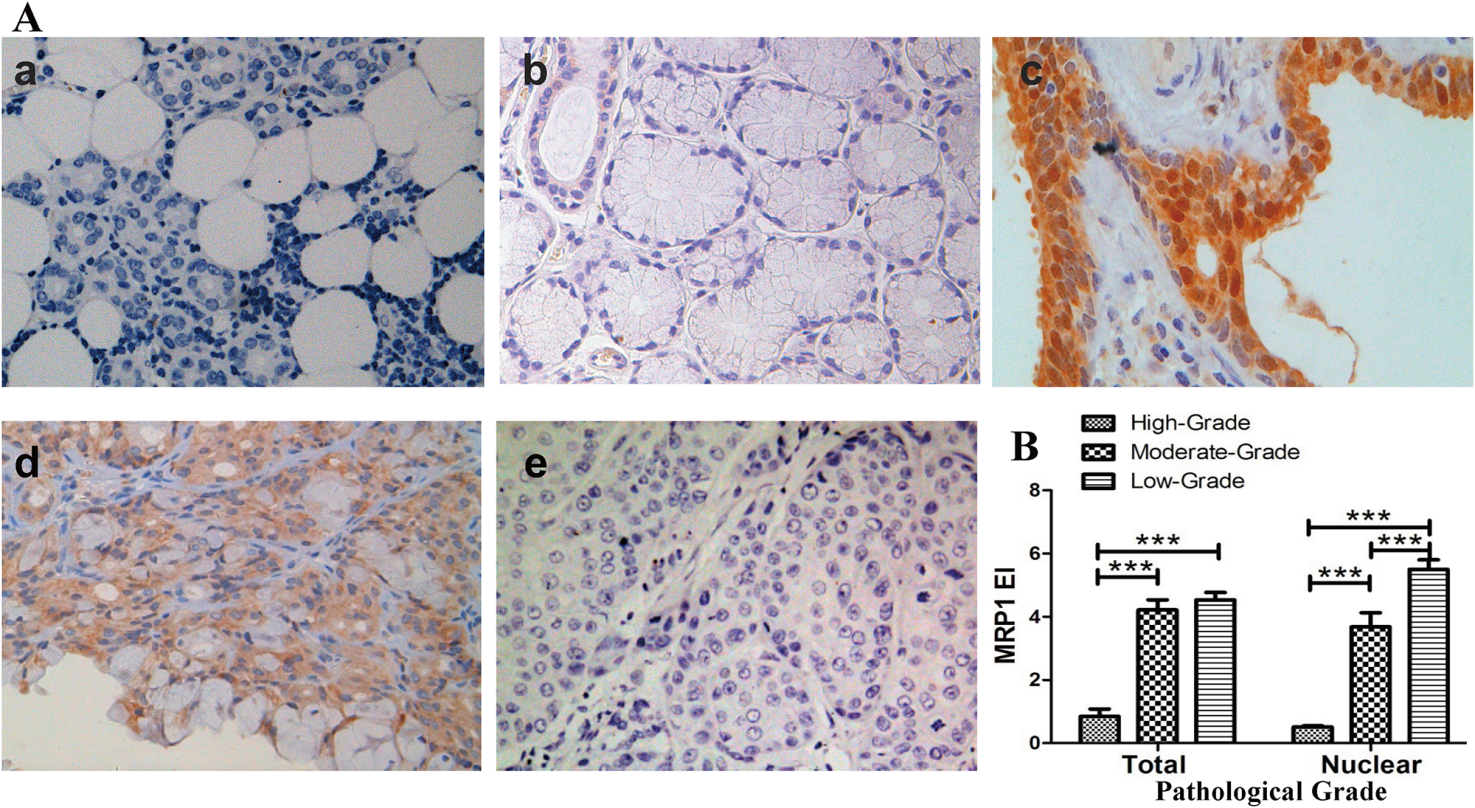
Nuclear MRP1 expression was negatively correlated to the pathologic grading of MEC. (A): a. No MRP1 was detected in normal salivary specimens; b. the expression of MRP1 in MEC adjacent tissues is slightly higher than normal tissues but still negligible, MRP1 was only lightly stained in the cells of striated duct; c. MRP1 was strongly expressed in the nuclei and lightly expressed in the cytoplasm in low-grade MEC; d. In moderate-grade MEC tissues, MRP1 was mostly distributed in the cytoplasm and rarely expressed in the nuclei of the cells; e. In high-grade MEC tissues, MRP1 was negligible. (B): Compared with moderate-grade or low-grade MEC, total MRP1 expression of high-grade MEC was significantly lower. But the difference of total MRP1 expression between moderate-grade MEC and low-grade MEC was not significant. The nuclear MRP1 expression significantly decreased as the pathologic grade increased. The differences of nuclear MRP1 expression among different pathologic grades were all significant (P<0.001). ***P<0.001.

MEC tumors were pathologically graded into low grade, moderate grade and high grade according to their pathologic features. Then, the location and intensity of MRP1 in normal salivary tissues, MEC adjacent tissues and MEC tissues were analyzed after immunohistochemical staining. No MRP1 was detected in normal salivary specimens (Fig 1Aa); the expression of MRP1 in MEC adjacent tissues is higher than normal tissues but still negligible, MRP1 was only lightly stained in the cells of striated duct which function to modify the osmotic pressure of salivary fluid (Fig 1Ab). MRP1 was expressed primarily in the nuclei and partly in the cytoplasm of cells in low-grade MEC tissues (Fig 1Ac). In moderate-grade MEC tissues, MRP1 was mostly distributed in the cytoplasm and rarely expressed in the nuclei of the cells (Fig 1Ad). In the high-grade cases, staining was negligible in both cytoplasm and nuclei of the cells (Fig 1Ae).

The expression index (EI) of total MRP1/nuclear MRP1 in tissue samples were calculated then statistically analyzed. The EI was scored relative to the healthy salivary acini as we described before [15]. Compared with moderate-grade (P<0.001) and low-grade (P<0.001) MEC, the expression level of total MRP1 appeared to be significantly lower in high-grade MEC patients. But no significant difference of total MRP1 expression was found between the moderate-grade and low-grade MEC. Interestingly, the nuclear MRP1 expression significantly decreased as the pathologic tumor grade increased. The nuclear MRP1 expression between each two of the pathologic grades was significantly different (P<0.001) (Fig 1B) (Table 1). The results above implied that the expression of nuclear MRP1 decreased as the malignant grade of MEC increased.

**Table 1:** Clinicopathologic parameters and the expression status of total MRP1/nuclear MRP1.

Characterstics	Number of cases (%)	Total MRP1 expression index:Mean±SE	P	Nuclear MRP1 expression index:Mean±SE	P
**Total number**	125				
**Age (years)**			0.95		0.95
≤40	50 (40%)	4.05±0.28		4.45±0.40	
>40	75 (60%)	4.01±0.27		4.49±0.35	
**Gender**			0.37		0.91
Female	71 (56.8%)	4.22±0.27		4.50±0.35	
Male	54 (43.2%)	3.87±0.28		4.44±0.41	
**Site**			0.44		0.67
Major glands	64 (51.2%)	4.22±0.27		4.37±0.37	
Minor glands	61 (48.8%)	3.91±0.29		4.59±0.39	
**Pathological Grade**			<0.001		<0.001
High	13 (10.4%)	0.85±0.23	***	0.52 ±0.33	***
Moderate	35 (28%)	4.22 ±0.32		3.68±0.45	
Low	77 (61.6%)	4.54±0.23		5.50±0.30	
**Tumor Stage**			0.18		0.014
T1	61 (48.8%)	4.39±0.27		5.08±0.36	*
T2	46 (36.8%)	4.03±0.33		4.39±0.43	
T3	13 (10.4%)	3.09±0.59		3.09±0.89	
T4	5 (4%)	3.12±1.18		1.52±0.53	
**Nodal Stage**			0.13		<0.001
N0	110 (88%)	4.18±0.21		4.82±0.28	***
N1	10 (8%)	3.81±0.58		2.60±0.77	
N2	5 (4%)	2.18±1.21		0.60±0.36	
**Distant Metastasis**					
M0	122 (97.6%)	4.17±2.12		4.5861±2.92	
M1	3 (2.4%)	0±0		0±0	
**Clinical Stage**			<0.01		<0.001
I	53 (42.4%)	4.55 ± 0.28	**	5.47 ± 0.37	***
II	41 (32.8%)	4.20 ± 0.34		4.76 ± 0.44	
III	21 (16.8%)	3.59 ± 0.42		3.12 ± 0.62	
IV	10 (8%)	2.01 ± 0.72		0.86 ± 0.34	

χ^2^ test, two sided;

*P<0.05,

**P<0.01,

***P<0.001.

The correlation between the EI of total/nuclear MRP1 and the pathologic grade of the MEC tissues was analyzed using Spearman’s rank correlation analysis. A negative correlation between total MRP1 expression and pathologic grade was found (r = -0.35, p<0.01). Furthermore, a stronger negative correlation was found between pathologic grade and nuclear MRP1 in MEC tissues (r = -0.498, p<0.01).

### 3.2 In low and moderate grade MEC, the nuclear MRP1 expression in MEC patients with lymphatic metastasis is significantly less than that in patients without lymphatic metastasis

In each pathologic grade, we compared the total/nuclear MRP1 expression in patients exhibiting no metastasis (MEC-NM) with the patients suffering lymph node metastasis (MEC-M). The metastasis rate was 38.5% in high grade MEC patients, no significant difference of total MRP1 expression (P = 0.08) or nuclear MRP1 expression (P = 0.24) was found between MEC-NM and MEC-M. The metastasis rate was 17.1% in moderate grade MEC patients, no significant difference of total MRP1 expression (P = 0.32) was found between MEC-NM and MEC-M, but a significant difference of nuclear MRP1expression (P = 0.012) was found between MEC-NM and MEC-M. The metastasis rate was 6.5% in low grade MEC patients, no significant difference of total MRP1 expression (P = 0.62) was found between MEC-NM and MEC-M, but a significant difference of nuclear MRP1 expression (P = 0.012) was found between MEC-NM and MEC-M (Table 2). Results above suggested that nuclear MRP1 could be a reference marker used to predict lymph node metastasis in MEC patients.

**Table 2:** Metastasis status and total/nuclear MRP1expression status in each pathologic grade.

Characterstics	Number of cases(%)	Total MRP1 expression index:Mean±SE	P	Nuclear MRP1 expression index:Mean±SE	P
**High Grade**	**13**		**0.08**		**0.24**
**Distant and lymphatic metastasis**	**5 (38.5%)**	**0.36±0.34**		**0.02±0.02**	
**Non-metastasis**	**8 (61.5%)**	**1.18±0.26**		**0.84±0.51**	
**Moderate Grade**	**35**		**0.32**		**0.012**
**Lymphatic metastasis**	**6 (17.1%)**	**3.50±0.77**		**1.27±0.65**	*
**Non-metastasis**	**29 (82.9%)**	**4.37±0.36**		**4.18±0.48**	
**Low Grade**	**77**		**0.62**		**0.012**
**Lymphatic metastasis**	**5 (6.5%)**	**4.10±0.90**		**2.66±0.44**	*
**Non-metastasis**	**72 (93.5%)**	**4.57±0.24**		**5.70 ±0.31**	

Student’s t-test;

*P<0.05.

### 3.3 Correlation between total/nuclear MRP1 expression and clinicopathological parameters of MEC patients

We examined the correlation between total/nuclear MRP1 expression and the clinicopathological parameters of the patients. No significant difference of total/nuclear MRP1 expression was observed between age, gender or tumor site (Table 1).

The correlation between the total/nuclear MRP1 expression and the clinicopathological staging of the MEC patients was analyzed using Spearman’s rank correlation analysis. No significant correlation was found between the total MRP1 expression and tumor staging (r = -0.18, P = 0.05), but an obvious negative correlation was found between nuclear MRP1 expression and tumor staging (r = -0.28, P = 0.02) in MEC patients. No significant correlation was found between total MRP1 expression and nodal staging (r = -0.13, P = 0.15), but an obvious negative correlation was found between nuclear MRP1 expression and nodal staging (r = -0.29, P<0.01) in MEC patients. A negative correlation between total MRP1 expression and clinical staging was found in MEC patients (r = -0.26, p<0.01). Furthermore, a stronger negative correlation was found between clinical staging and nuclear MRP1 expression in MEC patients (r = -0.41, p<0.01).

**Fig 2:**
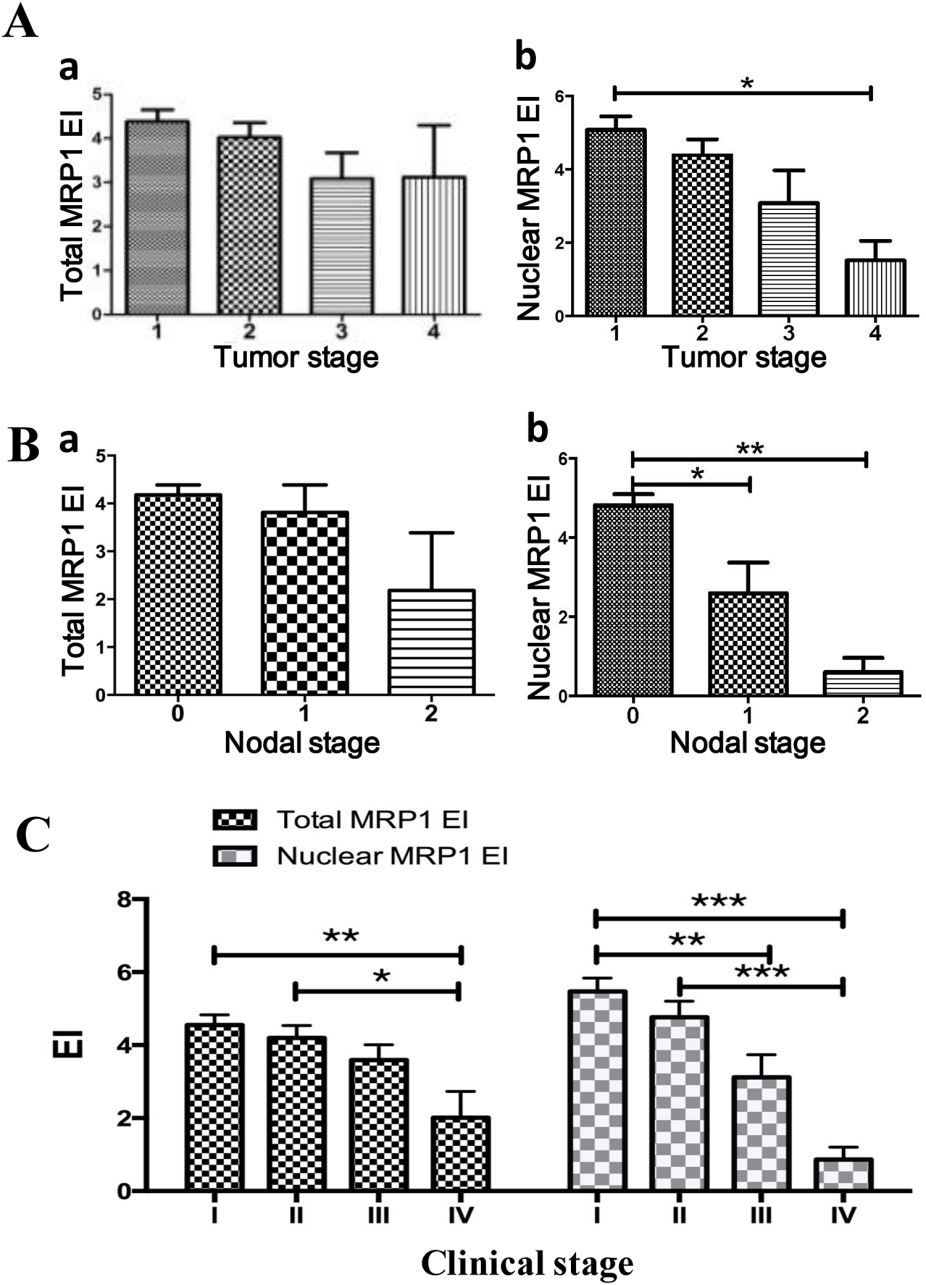
Nuclear MRP1 expression was negatively correlated to the clinical staging of MEC. (A) a. The difference of total MRP1 expression among different tumor stages (P = 0.18) was not significant; b. The expression of nuclear MRP1 decreased as the T stage upgraded. Furthermore, nuclear MRP1 expression in T1 was significantly higher than that in T4. (B) a. The total MRP1 expression among different nodal stages was not significantly different (P = 0.13); b. The nuclear MRP1 expression in N0 was significantly higher than that in N1 (P<0.05) and N2 (P<0.01). (C) Total MRP1 expression between stage 1 and stage 4 (P<0.01), between stage 2 and stage 4 (P<0.05) were significantly different. Nuclear MRP1 expression among different clinical stages was significantly different (P<0.001). Nuclear MRP1 expression between stage 1 and stage 4 (P<0.001), between stage 1 and stage 3 (P<0.01), between stage 2 and stage 4 (P<0.01) were all significantly different. *P<0.05, **P<0.01, ***P<0.001.

The total MRP1 expression among different tumor stages (T stage) (P = 0.18) (Fig 2Aa) or the nodal stages (N stage) (P = 0.13) (Fig 2Ba) is not significantly different. However, the nuclear MRP1 expression among different T stages (P = 0.014) is significantly different. We found the expression of total MRP1 decreased as the tumor size increased, and the total MRP1 expression in T1 was significantly higher than in T4 (P<0.05) (Fig 2Ab). The nuclear MRP1 expression among different N stages (P<0.01) is also significantly different. The nuclear MRP1 expression in N0 is significantly higher than N1 (P<0.05). Also, the nuclear MRP1 expression in N0 is significantly higher than N2 (P<0.01) (Fig 2Bb). The results suggested that the less the nuclear MRP1 was expressed, the more the lymph node metastasis happened. Three MEC patients suffered distant metastasis in our study, but the total/nuclear MRP1 expression in their MEC tissues was negligible (EI = 0) which rendered the statistic results unreliable (Table 1).

The total MRP1 expression among different clinical stages is significantly different (P<0.01). The total MRP1 expression between stage 1 and stage 4 (P<0.01) and between stage 2 and stage 4 (P<0.05) is significantly different. The nuclear MRP1 expression among different clinical stages is significantly different (P<0.001). The nuclear MRP1 expression between stage 1 and stage 4 (P<0.001), between stage 1 and stage 3 (P<0.01) and between stage 2 and stage 4 (P<0.01) is also significantly different (Fig 2C).

### 3.4 Down-regulation of nuclear MRP1 motivated the proliferation of MEC cells

In this study, we found nuclear MRP1 was highly related to the pathologic grade and clinical stage of MEC. The results above indicated that nuclear MRP1 may contribute to the progression of MEC. To verify the roles of nuclear MRP1 in MEC, the effects of RNAi-mediated downregulation of nuclear MRP1 on MEC cells were assessed. The expression of MRP1 was down-regulated by short-hairpin RNA (shRNA). The multidrug-resistant MC3/5FU cells were transfected with plasmids containing a MRP1 specific shRNA and a non-specific control shRNA. The resulting clones were MC3/5FU—S and MC3/5FU-NS. The down-regulation of nuclear MRP1 was verified by RT-PCR, western blot analysis and immunofluorescent confocal laser-microscopy in our previous study [16].

**Fig 3:**
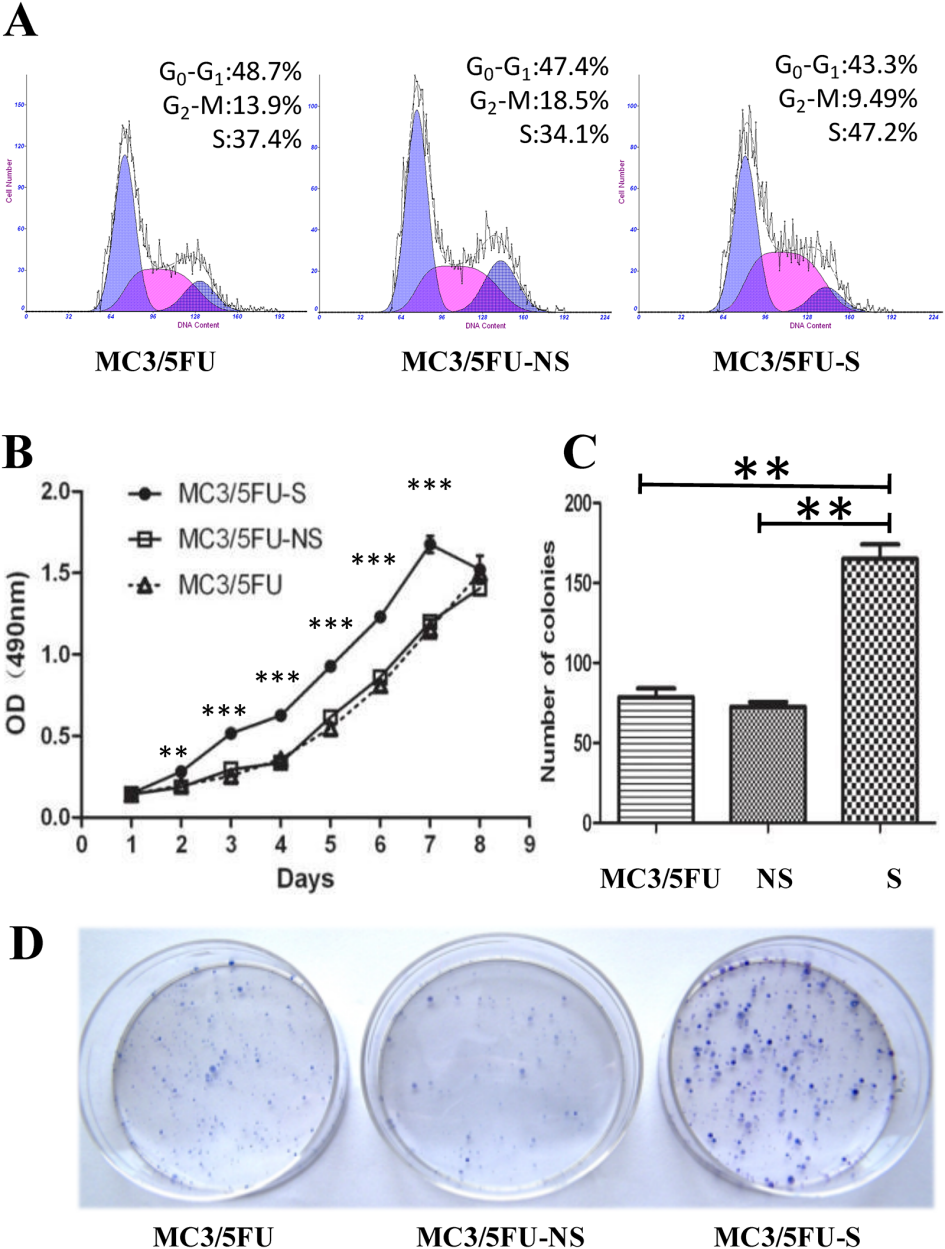
Down-regulation of nuclear MRP1 motivated the growth of MEC cells. The expression of nuclear MRP1 is down-regulated by short-hairpin RNA (shRNA). The multidrug-resistant MC3/5FU cells were transfected with plasmids containing an MRP1 specific shRNA and a non-specific control shRNA, the resulting clones were MC3/5FU—S (short for S) and MC3/5FU-NS (short for NS). (A) Flow cytometry was used to determine the altered cell cycle of MEC cells as nuclear MRP1 decreased. Compared with MC3/5FU cells (49.9±1.01%) and MC3/5FU-NS cells (48.76±0.63%), the percentage of MC3/5FU-S cells in G0/G1 phase significantly decreased to 44.03±1.04% (P<0.01, n = 4). Compared with MC3/5FU (15.25±1.25%) and MC3/5FU-NS (16.61±1.04%), the percentage of MC3/5FU-S cells in G2/M phase was also significantly decreased to 11.20±0.77% (P<0.05, n = 4). Also compared with MC3/5FU (34.70±1.19%) and MC3/5FU-NS (34.26±0.63%), the cell number of MC3/5FU-S cells in S phase significantly increased to 43.63±1.49% (P<0.01, n = 4). (B) The growth curves showed that the viability of MC3/5FU-S cells was obviously higher than that of MC3/5FU cells and MC3/5FU-NS cells during the exponential growth phase between day 2 and day 7. (C) Compared with MC3/5FU (78.67±5.49) and MC3/5FU-NS cells (72.67±2.91), plate colony formation assay showed that colony number of MC3/5FU-S (165.0±9.07) cells substantially increased (P<0.01, n = 3). (D) After 14 days of culturing, the colonies of MEC cells were fixed then stained. The staining showed that not only the number of MC3/5FU-S colonies was obviously more substantial than other groups but also the size of the MC3/5FU-S colonies was larger in relation to other groups. **P<0.01, ***P<0.001.

Flow cytometry was used to determine the altered cell cycle of MEC cells. Compared with MC3/5FU cells (49.9±1.01%) and MC3/5FU-NS cells (48.76±0.63%), the percentage of MC3/5FU-S cells in G0/G1 phase significantly decreased to 44.03±1.04% (P<0.01, n = 4). Compared with MC3/5FU (15.25±1.25%) and MC3/5FU-NS (16.61±1.04%), the percentage of MC3/5FU-S cells in G2/M phase was also significantly decreased to 11.20±0.77% (P<0.05, n = 4). Lastly, compared with MC3/5FU (34.70±1.19%) and MC3/5FU-NS (34.26±0.63%), the cell number of MC3/5FU-S cells in S phase significantly increased to 43.63±1.49% (P<0.01, n = 4) (Fig 3A).

The growth curves showed that the OD (490 nm) of MC3/5FU-S cells was obviously higher than that of MC3/5FU cells or MC3/5FU-NS cells during the exponential growth phase between day 2 and day 7 (Fig 3B). Plate colony formation assay showed that the colony number of MC3/5FU-S (165.0±9.07) cells was significantly more than MC3/5FU (78.67±5.49) cells and MC3/5FU-NS cells (72.67±2.91) (P<0.01, n = 3) (Fig 3C). We also found the size of the MC3/5FU-S colonies was bigger than the colonies of the other two groups (Fig 3D). Results above indicated that nuclear MRP1 may impact on the growth and colony formation via regulating cell cycle.

### 3.5 Down-regulation of nuclear MRP1 enhanced the migration and invasion ability of MEC cells

**Fig 4:**
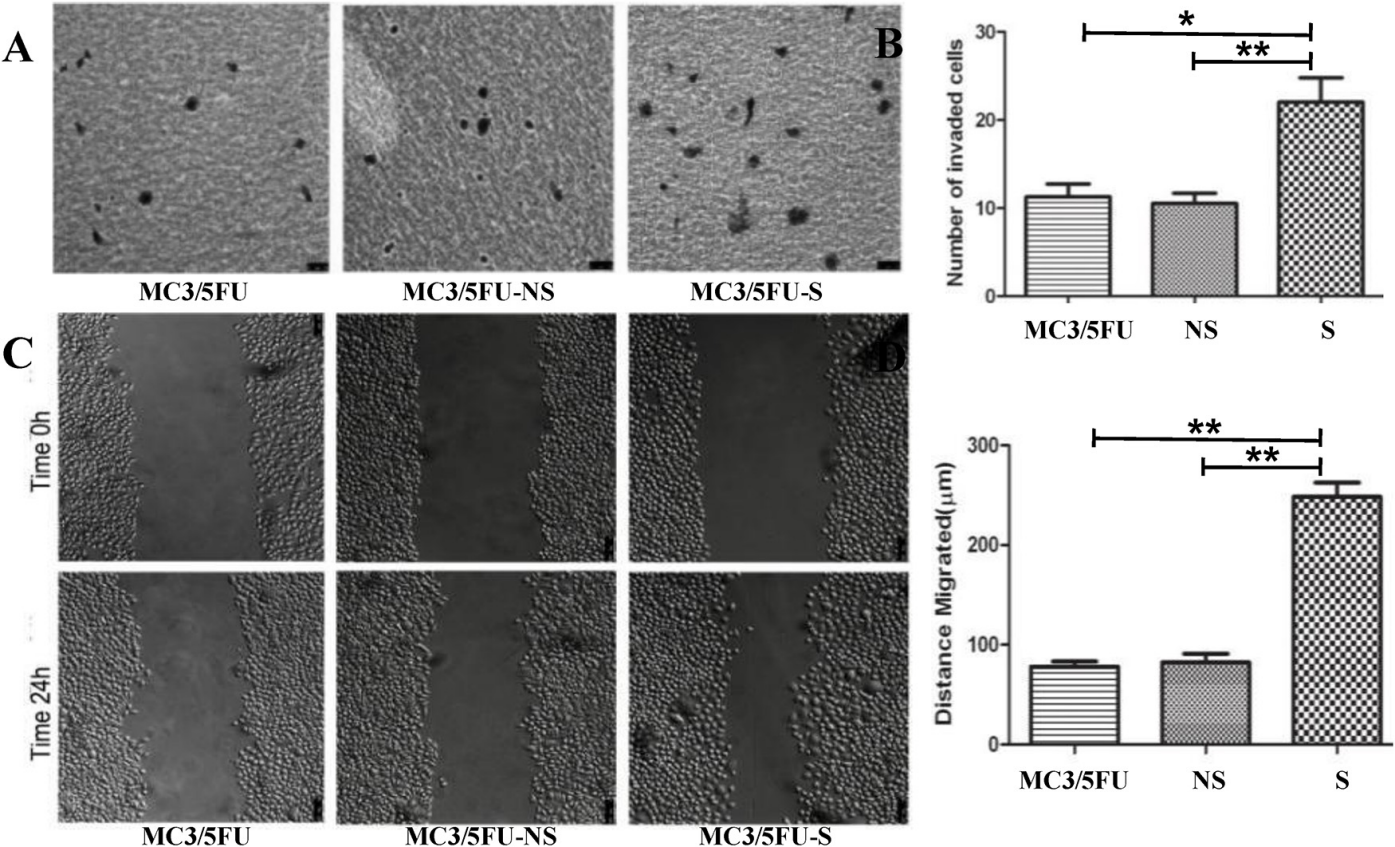
Down-regulation of nuclear MRP1 increased the migration and invasion of MC3/5FU cells. (A) The cells that migrated to the lower surface of the filter were stained with Giemsa. The Transwell invasion assay showed that more MC3/5FU-S cells invaded through the filter than the other two groups. (B) The average number of cells in 6 high power fields was 22.0±2.80 for the MC3/5FU-S cells, which is significantly higher than the MC3/5FU cells (11.25±1.49, P = 0.015, n = 3) and the MC3/5FU-NS cells (0.50±1.19, P<0.01, n = 3). No significant difference was found between MC3/5FU cells and MC3/5FU-NS cells. (C)The migration distance in the wound was calculated by the formula below: cell free area at 0 hour—cell free area at 24 hour. (D) The migration distance of MC3/5FU-S cells was 257.0±21.44μm, which is significantly higher than that of MC3/5FU cells (78.09±5.29μm, P<0.01, n = 3) and the MC3/5FU-NS cells (2.29±8.84μm, P<0.01, n = 3). No significant difference was found between MC3/5FU cells and MC3/5FU-NS cells.

In transwell invasion assays, the average amount of MC3/5FU-S cells, which crossed the matrigel-coated membrane, was 22±2.80, significantly higher than that of MC3/5FU cells (11.25±1.49, P = 0.015, n = 3) and MC3/5FU-NS cells (10.50±1.19, P<0.01, n = 3) (Fig 4A and 4B). In monolayer wound healing assays, the migration distance of MC3/5FU-S cells was 257.0±21.44μm, significantly higher than that of the MC3/5FU cells (78.09±5.29μm, P<0.01, n = 3) and the MC3/5FU-NS cells (2.29±8.84μm, P<0.01, n = 3) (Fig 4C and 4D).

## Disscusion

For now, radical surgery, lymphadenectomy and adjuvant external beam radiotherapy are the typical therapeutic options for MEC treatment. Chemotherapy is mainly used for palliative treatment of metastatic disease only [19,20]. As the traditional treatment has now reached a bottleneck, the exploration of novel biomarkers and targeted therapies of mucoepidermoid carcinoma (MEC) becomes an imperative endeavor in extending patients’ prognosis.

At present, the pathologic grade is the most accepted prognosis predictor used to guide treatment of mucoepidermoid carcinoma [20–22]. As the pathologic grade is upgraded, atypical mitosis, anaplasia, infiltrative border, necrosis, perineural and angiolymphatic invasion are more common, the recurrence rate and metastasis rate increased simultaneously [19,23,24]. T stage reflected the tumor size, as well as tumor invasion depth. In this study, we found the expression of nuclear MRP1 decreased as the tumor pathologic grade and the T stage increased (Figs 1 and 2Ab). The Spearman’s rank correlation analysis also provided evidence that the nuclear MRP1 expression was significantly negatively correlated with the pathologic grade and T stage of MEC. The RNA interference study further proved that nuclear MRP1 could suppress the proliferation and invasion of MEC cells, induce cell cycle arrest of MEC cells (Fig 3). Results above reflected that nuclear MRP1 was a prominent prognostic marker of MEC.

It’s pointed out that lymph nodes metastasis of MEC is the strongest prognostic factor of treatment failure [13,14]. Our results indicated that nuclear MRP1 decreased as the N stage (lymph node metastasis) increased (Fig 2Bb). Even in the same pathologic grade, the nuclear MRP1 expression in MEC patients with lymphatic metastasis is obviously less than that in patients without lymphatic metastasis (Table 2). The Spearman’s rank correlation analysis also provided evidence that the nuclear MRP1 expression was significantly negatively correlated with N stage (lymph node metastasis) of MEC. The RNA interference study showed further evidence that nuclear MRP1 was able to suppress the invasion and migration ability of MEC cells in vitro. Furthermore, invasion from primary tumor site and target migration are two key steps in whole process of metastasis [25]. It’s known that patients with MEC generally carry a high risk of lymph node metastases [13]. However, because of the tremendous trauma caused by neck dissection, doctors prudently choose to perform neck dissection on MEC patients, even though lymph node involvement means terrible prognosis [26]. In the standard treatment procedure of MEC, neck dissection is required only when lymph node infiltration is found. However, current examinations are unable to detect cell-level metastasis, leading to a high probability for future treatment failure. Our findings showed that nuclear MRP1 could be a prognostic marker to predict the metastasis of MEC.

It is generally accepted that MRP1 is an energy-dependent transporter and overexpression of MRP1 is a predictor of poor response to chemotherapy in a variety of hematological and solid tumors [2]. However, the prognostic function of MRP1 in tumor patients without pretreatment of chemotherapy is not well studied. We found that the studies about the prognostic value of MRP1 were mainly focused on advanced tumors, however, patients included in our investigation mainly suffered from early stage tumors. Further more, most of the researches were focused on the fact that downregulation of MRP1 could decrease the multidrug-resistance of tumor cells. However, no obvious evidence has been found that downregulation of MRP1 would increase the proliferation, invasion or migration of tumor cells. On the contrary, it was reported that MRP1-knockout mice becomes more fertile and shows enhanced reendothelialization after vascular injury [5]. Furthermore, it was also reported that MRP1 overexpressing tumors are less aggressive and more differentiated, and the overexpression of MRP1 is correlated with better prognosis in the non-small cell lung patients without chemotherapy [3,4]. While upon initial observation, the facts about MRP1appear to be conflicted, but this paradox merely reflects the key role of MRP1 in maintaining cellular environmental homeostasis.

Our previous study found that nuclear translocation of MRP1 contributed to the multidrug-resistance by suppressing the apoptosis of MEC cells. Then, we proved that the nuclear MRP1could regulate the activity of the promoter of MDR1 in the nucleus [15,16]. We thought nuclear MRP1 participated in numerous cell signaling processes by altering the glutathione (GSH) content in the nucleus. GSH is the most important redox agent and antidote in the cells which decided the redox state of the nucleus. By affecting the redox state of the nucleus, GSH participates in numerous cell-signalling processes including DNA repair, cell circles and cell suicide programs [27,28]. MRP1, as the main transporter of GSH, participates in numerous metabolic and cell signaling processes though modulating GSH content in the cells [28]. Immunohistochemical staining was conducted to check the localization and expression of several extensively concerned MDR-related proteins: Lung resistance protein (LRP), Topoisomerase II (Topo II) and glutathione-S-transferase-π (GST-π). The results showed that the expression of glutathione-S-transferase-π (GST-π) in MEC tissues was obviously higher than normal salivary gland tissues (S1 Fig). This result partly supported our previous hypothesis that when chemotherapeutic drugs were added, nuclear MRP1 maintained the basic function of nucleus by transporting GSH from cytoplasm into nucleus and GST-π aided in detoxification by catalyzing the conjugation of a wide number of exogenous and endogenous hydrophobic electrophiles with reduced glutathione [29]. In the study of MEC patients without receiving any pretreatment before surgeries, we found that nuclear MRP1 decreased as the pathologic grade and clinical stage upgraded. The RNA interference study also proved that the downregulation of nuclear MRP1 increased the proliferation rate, invasion and metastasis of MEC cells (Fig 4A–4D). We thought that nuclear MRP1 participated in numerous cell signaling processes by transporting glutathione (GSH) into nucleus. When chemotherapeutic drugs were absent, the nuclear MRP1 would continue on decreasing cytoplasmic GSH in the MEC cells, thereby activating p38MAPK pathways [30]. The activation of p38 may be the reason that nuclear MRP1 enhances the chemo-resistance of MEC but suppresses its proliferation and metastasis of MEC cells [31,32].

## Conclusions

In conclusion, our results suggested that nuclear MRP1 is a promising biomarker associated with better prognosis of MEC and further study of its function mechanism would provide clues in developing new treatment modalities of MEC.

## Supporting Information

S1 Fig Lung resistance protein (LRP), Topoisomerase II (Topo II) and glutathione-S-transferase Pi (GST-π) expression in normal salivary gland tissues (SG) and mucoepidermoid carcinoma (MEC) tissues. LRP has been found to be the major component of vaults, and considered to mediate drug redistribution by regulating both cytoplasmic and nucleo-cytoplasmic transport. It has been reported that LRP is correlated with resistance to anticancer drugs such as etoposide, doxorubicin and paclitaxel, but also to nonclassical MDR drugs such as cisplatin and carboplatin. Topoisomerase II (Topo II) are ubiquitously expressed enzymes which can result from DNA replication, transcription and repair. Topoisomerase II has therefore become the main target of many antitumor therapy regimens, even though the exact mechanism of cell killing remains elusive. In mammals, there are six different cytosolic glutathione-S-transferase (GST) isoforms: alpha, mu, pi, theta, omega, and zeta. GST-π (Pi) is of particular interest with regard to cancer, because many tumors and cancer cell lines are characterized by high GST-Pi expression. Further, increased expression of GST-π has also been linked to acquired resistance to cancer drugs. Immunohistochemical staining was conducted to determine the localization and expression index (EI) of LRP, TOPO-II and GST-π in formalin-fixed, paraffin-embedded MEC tissues and normal salivary gland tissues. (a, d): The expression of glutathione-S-transferase Pi (GST-π) in MEC tissues was obviously higher than normal salivary gland tissues (Rebuttal Fig 3). (b, e): No obvious difference of LRP expression was found between MEC tissues and normal salivary gland tissues. (c, f): No obvious difference of Topo-II expression was found between MEC tissues and normal salivary gland tissues. Lung resistance protein (LRP), Topoisomerase II (Topo II) and are all extensively concerned MDR-related proteins.
(DOCX)
