# Supplementary material for: Nuclear Multidrug Resistance-Related Protein 1 Is Highly Associated with Better Prognosis of Human Mucoepidermoid Carcinoma through the Suppression of Cell Proliferation, Migration and Invasion
Source: PLoS One. 2016 Feb 1;11(2):e0148223. doi: 10.1371/journal.pone.0148223 (PMC4734599; doi:10.1371/journal.pone.0148223)
Supplement: S1 Fig — LRP has been found to be the major component of vaults, and considered to mediate drug redistribution by regulating both cytoplasmic and nucleo-cytoplasmic transport. It has been reported that LRP is correlated with resistance to anticancer drugs such as etoposide, doxorubicin and paclitaxel, but also to nonclassical MDR drugs such as cisplatin and carboplatin. Topoisomerase II (Topo II) are ubiquitously expressed enzymes which can result from DNA replication, transcription and repair. Topoisomerase II has therefore become the main target of many antitumor therapy regimens, even though the exact mechanism of cell killing remains elusive. In mammals, there are six different cytosolic glutathione-S-transferase (GST) isoforms: alpha, mu, pi, theta, omega, and zeta. GST-π (Pi) is of particular interest with regard to cancer, because many tumors and cancer cell lines are characterized by high GST-Pi expression. Further, increased expression of GST-π has also been linked to acquired resistance to cancer drugs. Immunohistochemical staining was conducted to determine the localization and expression index (EI) of LRP, TOPO-II and GST-π in formalin-fixed, paraffin-embedded MEC tissues and normal salivary gland tissues. (a, d): The expression of glutathione-S-transferase Pi (GST-π) in MEC tissues was obviously higher than normal salivary gland tissues (Rebuttal Fig 3). (b, e): No obvious difference of LRP expression was found between MEC tissues and normal salivary gland tissues. (c, f): No obvious difference of Topo-II expression was found between MEC tissues and normal salivary gland tissues. Lung resistance protein (LRP), Topoisomerase II (Topo II) and are all extensively concerned MDR-related proteins. (DOCX) [file pone.0148223.s001.docx]

**Supporting Information**

**Immunohistochemistry**

Four normal salivary gland tissues (SG) and four salivary gland mucoepidermoid carcinoma (MEC) tissues were collected from Department of Oral and Maxillofacial Surgery, School of Stomatology, The Fourth Military Medical University, Xi’an, PR China. After being formalin-fixed, paraffin-embedded, the specimens were diagnosed and identified by the Department of Oral Histology and Pathology, School of Stomatology, Fourth Military Medical University, Xi’an, PR China. Sections for glutathione-S-transferase Pi (GST-π), Lung resistance protein (LRP), Topoisomerase II (TOPO-II), staining were pretreated by boiling in Tris/EDTA (1 mmol/l in distilled water, pH 9.0, Zhongshan, Beijing, China) for 20 minutes, sections for MDR1 staining were pretreated by boiling in citrate buffer (0.01 mol/l in distilled water, pH 6.0, Zhongshan, Beijing, China) for 2 min in a steaming pressure cooker. Immunohistochemistry assay (IHC) was performed with Streptavidin Biotin Peroxidase Detection kit from Zhongshan (Beijing, China) according to the manufacturer’s protocol. Rabbit anti-TOPO-II, as the primary antibody, was obtained from Santa Cruz, CA (1:50). Rabbit anti-GST-π, as the primary antibody, was obtained from Santa Cruz, CA (1:250). Rabbit anti-LRP, as the primary antibody, was obtained from Santa Cruz, CA (1:100). The slides were stained with 3,3’-diaminobenzidine (DAB) and counterstained with hematoxylin, then observed under a microscope (Leica DMI6000 B Fully Automated Inverted Research Microscope, Germany) with 100× magnification.

**S1 Fig. Lung resistance protein (LRP), Topoisomerase II** **(Topo II) and glutathione-S-transferase Pi (GST-π) expression in normal salivary gland tissues (SG) and mucoepidermoid carcinoma (MEC) tissues**

LRP has been found to be the major component of vaults, and considered to mediate drug redistribution by regulating both cytoplasmic and nucleo-cytoplasmic transport [1]. It has been reported that LRP is correlated with resistance to anticancer drugs such as etoposide, doxorubicin and paclitaxel, but also to nonclassical MDR drugs such as cisplatin and carboplatin [2]. Topoisomerase II (Topo II) are ubiquitously expressed enzymes which can result from DNA replication, transcription and repair [3]. Topoisomerase II has therefore become the main target of many antitumor therapy regimens, even though the exact mechanism of cell killing remains elusive. In mammals, there are six different cytosolic glutathione-S-transferase (GST) isoforms: alpha, mu, pi, theta, omega, and zeta. GST-π (Pi) is of particular interest with regard to cancer, because many tumors and cancer cell lines are characterized by high GST-Pi expression. Further, increased expression of GST-π has also been linked to acquired resistance to cancer drugs [4]. Immunohistochemical staining was conducted to determine the localization and expression index (EI) of LRP, TOPO-II and GST-π in formalin-fixed, paraffin-embedded MEC tissues and normal salivary gland tissues. (a, d): The expression of glutathione-S-transferase Pi (GST-π) in MEC tissues was obviously higher than normal salivary gland tissues (Rebuttal Figure 3). (b, e): No obvious difference of LRP expression was found between MEC tissues and normal salivary gland tissues. (c, f): No obvious difference of Topo-II expression was found between MEC tissues and normal salivary gland tissues. Lung resistance protein (LRP), Topoisomerase II (Topo II) and are all extensively concerned MDR-related proteins.

**References**

1. Izquierdo MA, Scheffer GL, Schroeijers AB, de Jong MC, Scheper RJ (1998) Vault-related resistance to anticancer drugs determined by the expression of the major vault protein LRP. Cytotechnology 27: 137-148.

2. Marcos-Carcavilla A, Calvo JH, Gonzalez C, Serrano C, Moazami-Goudarzi K, et al. (2008) Structural and functional analysis of the ovine laminin receptor gene (RPSA): Possible involvement of the LRP/LR protein in scrapie response. Mamm Genome 19: 92-105.

3. Pilati P, Nitti D, Mocellin S (2012) Cancer resistance to type II topoisomerase inhibitors. Curr Med Chem 19: 3900-3906.

4. Tew KD, Townsend DM (2011) Regulatory functions of glutathione S-transferase P1-1 unrelated to detoxification. Drug Metab Rev 43: 179-193.
